# Median arcuate ligament syndrome masquerading as mesenteric angina

**DOI:** 10.1002/ccr3.4579

**Published:** 2021-08-06

**Authors:** Ammar Chapra, Neethu Maria Kunjumon, Abdel‐Naser Elzouki, Mohammed Ibn‐Mas'ud Danjuma

**Affiliations:** ^1^ Internal Medicine Residency Program Hamad Medical Corporation Doha Qatar; ^2^ Department of Internal Medicine Hamad General Hospital Doha Qatar; ^3^ Department of Clinical Medicine Qatar University Doha Qatar; ^4^ Weill Cornell Medicine Doha Qatar; ^5^ Clinical Pharmacology and Therapeutics Department of General Medicine Hamad General Hospital Doha Qatar; ^6^ Department of Clinical Medicine Weill Cornell Medicine Doha Qatar

**Keywords:** celiac artery compression syndrome, Dunbar syndrome, median arcuate ligament syndrome, mesenteric angina, recurrent abdominal pain

## Abstract

Patients with recurrent unclear causes of postprandial abdominal pain should have median arcuate ligament syndrome as a differential diagnosis which is thought to be caused by celiac artery compression. Diagnosis is by imaging such as CT angiography.

## Graphical abstract

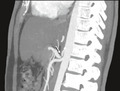

## 1 INTRODUCTION

Recurrent abdominal pain despite being a common reason for a visit to the Emergency Department (ED) can be fraught with diagnostic uncertainties. One such condition to keep in mind, which requires the need of extra vigilance during diagnostic workup for abdominal pain, is median arcuate ligament syndrome (MALS).

The diagnostic workup for abdominal pain presenting to the Emergency Department (ED) is sometimes fraught with considerable uncertainties.^1^ This is especially true for patients with recurrent presentations with abdominal pain and normal investigations. Causes of postprandial abdominal pain include mesenteric ischemia among others.^2^, ^3^ We present a case of postprandial abdominal pain due to median arcuate ligament syndrome.

## 2 CASE REPORT

A seventy‐one‐year‐old male patient of Middle‐Eastern, well known to the Emergency Department (ED) due to recurrent monthly visits with abdominal pain, presented to the ED with a similar presentation of greater severity for the past 1 day, along with few episodes of vomiting. On this visit, his pain was dull and diffuse, however, felt more intensely in the Right hypochondrium radiating all over the abdomen. Food intake consistently made the pain worse, and no relieving factors were known aside from immediate resolution of pain during prior ED visits with analgesic medications including intravenous (IV) opioids.

Review of his comorbidities included hypertension, type 2 diabetes mellitus, and dyslipidemia, all of which were well controlled with medications. He had a history of gastritis non‐responsive to proton pump inhibitor (PPI) therapy. He was known to physicians taking care of him in the past as a chronic case of irritable bowel syndrome refractory to medical management. Aside from these, he had history of a hiatal hernia, managed conservatively, and had undergone a laparoscopic appendectomy 10 years prior.

Physical examination similar to prior episodes revealed epigastric tenderness out of proportion to the severity of pain, and aside from this was unremarkable.

A routine panel of investigations recurrently ordered, aside from mild corrected hypercalcemia of 2.58 mmol/L, was entirely unremarkable including liver function tests and pancreatic enzymes. C‐reactive protein was mildly elevated at 10.7 mg/L.

Computed tomography (CT) of the abdomen was then performed which showed re‐demonstration of a narrowed segment in the proximal celiac artery with a hook‐like configuration due to indentation of the median arcuate ligament in the superior aspect of the celiac artery. The patient received IV Morphine and IV Esomeprazole and was admitted under the care of Internal Medicine. However, his pain improved, and he was discharged with a schedule to be seen in vascular surgery outpatient department.

A review of past records remarkably revealed that the patient had been frequently visiting the ED since the past 15 years with on average more than 10 visits annually with the same complaint of postprandial pain. The location would vary on each presentation but was consistently dull, diffuse and worsened with eating. It would occasionally be associated with episodes of vomiting and anorexia; however, there was no weight loss. Examination findings on each visit would reveal tenderness out of proportion to the pain experienced by the patient. Laboratory findings would be unremarkable; however, the lactic acid level during these visits was variable with a range of 0.8–5.2 mmol/L with a mean of above 2.0 mmol/L. His liver, pancreatic enzymes along with tumor markers, thyroid function tests, and autoimmune panel all failed to reveal any abnormality. He even underwent bowel screen for occult blood, which was negative.

Owing to the frequency of his presentations which at times would be twice in the same day, he underwent an exhaustive diagnostic workup over the past 15 years which included two gastroscopies which revealed mild gastritis each time with *Helicobacter pylori (H*.*pylori)* testing being positive once. However, its eradication using stool antigen testing was documented later on. Ultrasonography of the abdomen would be performed on each visit which would be unremarkable. His first CT abdomen (with contrast) was unable to reveal any evidence of celiac artery compression with no other radiological findings that would account for his recurrent abdominal pain.

**FIGURE 1:**
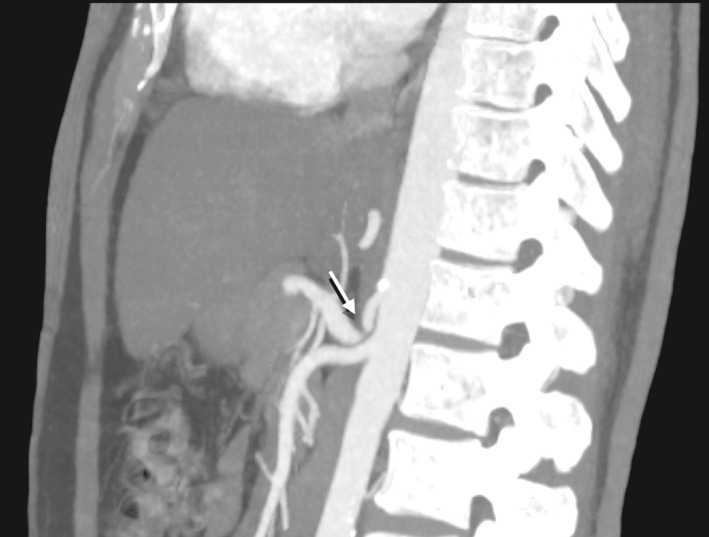
Computed tomography angiography (CTA) of the abdomen of the patient, parasagittal reconstruction showing a narrowed celiac artery with hook‐like configuration *(white arrow)* due to indentation of median arcuate ligament in the superior aspect of the celiac artery consistent with median arcuate ligament compression. Mild poststenotic dilatation of celiac artery can be visualized

Three years later, a second CT abdomen demonstrated the presence of a "hook"‐shaped configuration of the celiac trunk, with indentation on its superior surface and focal luminal narrowing at this level with mild poststenotic dilation of the celiac artery, concerning for median arcuate ligament syndrome. He was scheduled to be seen by the vascular surgery in an outpatient setting and was discharged after resolution of pain with IV analgesics, yet he missed his appointment. Subsequently, few months later, on one similar presentation of abdominal pain, CT angiography was done (Figure 1) suspecting mesenteric ischemia which confirmed the presence of median arcuate ligament compression on the celiac artery and ruled out any narrowing in superior and inferior mesenteric arteries.

Eventually, patient underwent a third CT abdomen that revealed no interval changes, and 3 months later, he presented to our care for the current presentation.

He was discharged with a follow‐up with vascular surgery for definitive management; however, he was lost to follow‐up.

## 3 DISCUSSION

The above case highlights the degree of uncertainty that precedes a diagnosis of median arcuate ligament syndrome (MALS). MALS or Dunbar syndrome as it is known since being described by Dunbar et al^4^ in 1965 as a constellation of abdominal symptoms which were since observed in several other cases as well. ^5^ Most common presenting complaints include epigastric pain, postprandial pain, nausea, vomiting, and weight loss.^5^ Considering a number of conditions could explain these rather vague symptoms, patients with MALS have usually had extensive workup done eventually leading to it being a diagnosis of exclusion.^6^

Owing to the fact that 40% of patients with MALS present with classic findings of abdominal angina, one of the proposed theories for its pathophysiology is compression of celiac trunk leading to mesenteric angina.^7^ Therefore, the diagnosis is critically dependent on radiological investigations such as open or laparoscopic angiography with both inspiratory and expiratory views most commonly used. However, computed tomography (CT) angiography or magnetic resonance angiography can also be utilized.^8^ A hook‐shaped configuration followed by dilatation of the celiac artery has classically been described.^7^

Despite the presence of typical findings in the presence of classic angiographic demonstration of celiac artery compression, the diagnosis of MALS may prove to be a diagnostic conundrum due to it being attributed to 2 out of 100,000 cases of upper abdominal pain.^9^ In addition to the above, 10%–24% of asymptomatic patients may demonstrate some degree of radiological compression by the median arcuate ligament.^6^ The uncertainty this produces may lead patients to be mislabeled despite having characteristic findings mentioned above, leading to secondary adverse outcomes such as analgesia dependency as evidenced by our case.

The mainstay of treatment is open, robotic, or laparoscopic ligament release, and although angioplasty remains an option, its role is limited by its inability to address the underlying compression resulting in the symptoms.^5^, ^7^

## 4 CONCLUSION

This case highlights the need for extra vigilance in the diagnostic workup of patients with recurrent presentations with unexplained abdominal pain. Comprehensive review of all radiological imaging of the abdomen may assist in providing the requisite diagnostic breakthrough including the possibility of syndromes such as MALS.

## CONFLICT OF INTEREST

None declared.

## AUTHOR CONTRIBUTIONS

AC involved in first author, manuscript writing, and literature review. NMK involved in case‐presentation writing. AE involved in literature review. MID involved in mentorship, manuscript writing, and literature review.

## ETHICAL APPROVAL

Ethical approval was obtained by Medical Research Center (MRC) under ID MRC‐04–19–436 on November 9, 2019.

## CONSENT STATEMENT

Published with written consent of the patient.

## Data Availability

All data generated during this study are included in this article.
